# Strengths use and thriving at work among nurses: a latent profile and mediation analysis

**DOI:** 10.1186/s12912-025-02715-8

**Published:** 2025-01-20

**Authors:** Jing Wu, Zhenrong Shen, Zidan Ouyang, Yuxuan Xiang, Ru Ding, Yuan Liao, Li Chen

**Affiliations:** 1 School of Nursing, Guangzhou University of Traditional Chinese Medicine, Guangzhou, Guangdong Province China; 2 The First Affiliated Hospital of Guangzhou University of Chinese Medicine, Guangzhou, Guangdong Province China

**Keywords:** Strengths use, Hope, Thriving at work, Nursing management

## Abstract

**Background:**

The ability to thrive at work has been demonstrated to be closely linked to the development of nurses.Effective utilization of strengths and maintaining hope are essential elements for clinical nurses’ ability to flourish in their roles. Nevertheless, the relationship between strengths use, hope, and thriving at work remains underexplored. This study aimed to identify distinct subgroups of clinical nurses based on their strengths use and to examine the mediating effect of hope between strengths use and thriving at work. The findings are intended to inform clinical managers on strategies to enhance nurses’ work performance and care quality.

**Methods:**

A convenience sample of 568 clinical nurses from two tertiary hospitals in Guangzhou City, Guangdong Province, China, was recruited between January and March 2024. The survey collected sociodemographic data and included the Adult Dispositional Hope Scale, Strengths Use Scale, and Thriving at Work Scale. Potential categories of nurse strengths use characteristics were identified using potential profile analysis, and potential relationships between variables were determined using Pearson correlation analysis, Bayesian factor robustness analyses, and Mediation analysis.

**Results:**

LPA identified three distinct groups based on strengths use: low (30.8%), moderate (64.9%), and high (13.2%).The significant mediating effect of hope in the relationship between strengths use based on latent profile analysis and thriving at work was observed (SE = 0.61,95%CI = -10.01, -7.62; SE = 0.76,95%CI = -18.91, -15.91, respectively).

**Conclusions:**

There exists heterogeneity in nurses’ strengths use. Hope plays a significant role in mediating the relationship between strengths use and thriving at work. It is recommended that nursing administrators or leaders pay attention to differences in individual levels of strengths use, encourage strengths-based practices and design interventions that foster hope, thereby promoting greater thriving in their professional roles.

## Introduction

Clinical nurses constitute a fundamental workforce within healthcare organizations, playing a central role in delivering patient care services [1]. Nonetheless, nurses all over the world are always under tremendous stress [2]. The demanding nature of hospital nursing, characterized by rapid workflow and substantial workloads, heightens nurses’ vulnerability to emotional exhaustion, contributing to elevated burnout levels and increased turnover intention [3]. Prior research has shown that flourishing can be a strategy to improve nurse retention [4].

Thriving at work is a psychological state characterized by both ‘learning’ and ‘vitality’ [5]. Nurses who thrive tend to exhibit lower absenteeism and turnover rates compared to those who do not, while also maintaining self-development and overall well-being, both physically and mentally [6, 7]. However, despite its sinificance, research indicates that nurses frequently encounter difficulties achieving this state, with consistently low vitality levels reported. For instance, a Belgian study found that nurses demonstrated moderately low vigor in their work [8]. Similarly, an online survey of 427 clinical nurses in China identified only moderate levels of thriving at work [9]. Understanding the factors that influence thriving could inform the development of targeted interventions aimed at improving nurses’ work conditions. Nonetheless, prior studies—primarily influenced by Spreitzer et al. [10] —have predominantly concentrated on external organizational factors, including leadership styles, workplace culture, and environmental conditions [11–13]. This focus has largely overlooked the pivotal role of intrinsic personal traits. Thus, the present study aims to expand the existing body of knowledge by investigating the fundamental individual-level prerequisites that contribute to thriving at work.

Focusing on leveraging personal strengths is widely regarded as an effective strategy to foster growth, development, and success [14]. Cross-cultural research reveals notable parallels in the virtues and strengths valued across societies, emphasizing that cultivating strengths is essential for realizing self-worth within a social context [15]. Strengths use, a central tenet of strengths theory, refers to the positive behaviors exhibited by employees when applying their strengths in the workplace [16]. Multiple studies indicate that strengths use addresses fundamental needs for autonomy, competence, and interpersonal relationships [17–19]. Self-determination theory posits that individuals are more inclined to engage in learning and participation when their intrinsic drives for growth and self-actualization are met [20, 21]. Engaging character strengths enhances individuals’ likelihood of thriving [22]. While interdisciplinary research has consistently shown a robust link between strengths use and flourishing at work [23, 24], empirical studies specifically targeting registered nurses remain limited, signifying a gap that requires further exploration. Moreover, previous research predominantly employs a variable-centered methodology to assess the degree of strengths use among nurses [25], which inadequately captures the diversity within and between groups. This limitation may reduce the efficacy of interventions aimed at enhancing strengths use. Latent profile analysis (LPA), a person-centered statistical method, classifies individuals based on shared personal and professional attributes, behaviors, or traits derived from their responses to specific observations [26]. The advantage of applying LPA in this study lies in its ability to deliver a more refined understanding of nurses’ strengths use by identifying distinct subgroups that could be overlooked in aggregate scoring. This approach provides valuable insights into the unique patterns of strengths use among nurses, informing both future research and the development of targeted interventions.

Hope, as a positive motivational state rooted in an inherent belief in success, encompasses willpower, goal-directed energy, and the strategies necessary to achieve goals [27]. Within the JD-R model, hope functions as a vital resource for addressing job demands, alleviating burnout, and sustaining job satisfaction among nurses [28, 29]. Its influence is particularly pronounced in fostering nurses' ability to thrive in the workplace. Initial results indicate that hope significantly predicts work flourishing among clinical nurses [30]. Furthermore, multiple studies highlight the substantial benefits of strengths-based approaches in cultivating elevated levels of hope [31–33]. For instance, research conducted on employees at the Centers for Disease Control and Prevention revealed a positive association between strengths use and hope [34]. The strengths perspective posits that individuals who identify, develop, and apply their strengths are more likely to engage proactively in goal achievement, which enhances their sense of satisfaction and accomplishment, which plays an important role in enabling nurses to excel mentally and at work [35]. This study aimed to examine the mediating role of hope in the relationship between strengths use and job thriving. Prior research has predominantly centered on the interaction between career and psychological capital [36–38], frequently integrating hope with constructs like optimism, self-efficacy, and resilience [39], while neglecting hope’s unique contributions. The relationship between strengths use, hope, and work flourishing remains underexplored.

The study aimed to investigate the relationship between nurses’ strengths use and workplace thriving, emphasizing the mediating role of hope. This research builds on existing literature in several key areas. Despite the growing interest in personal strengths, research specific to certain professions, such as nursing, remains limited. Additionally, most current studies, both nationally and internationally, tend to focus on group-level analyses, overlooking individual variability. This approach often fails to provide tailored guidance for personalized practice. To address this, latent class analysis was employed to identify heterogeneity in strengths use among nurses. Furthermore, while existing evidence on the link between healthcare workers’ strengths use and thriving is inconclusive, this study explored this relationship within the context of Chinese cultural norms, thereby increasing the ecological validity. Moreover, although prior research suggests strong associations between personal strengths, hope, and job thriving, these variables have rarely been examined in conjunction. This study employed mediation models to analyze hope as a mediator between strengths use and job flourishing. Based on these objectives and the theoretical framework, three hypotheses were proposed:

- H1: Strengths use is significantly associated with thriving at work;
- H2: The heterogeneity of strengths use can be identified through LPA;
- H3: Hope mediates the relationship between strengths use and thriving at work.

**Fig. 1:**
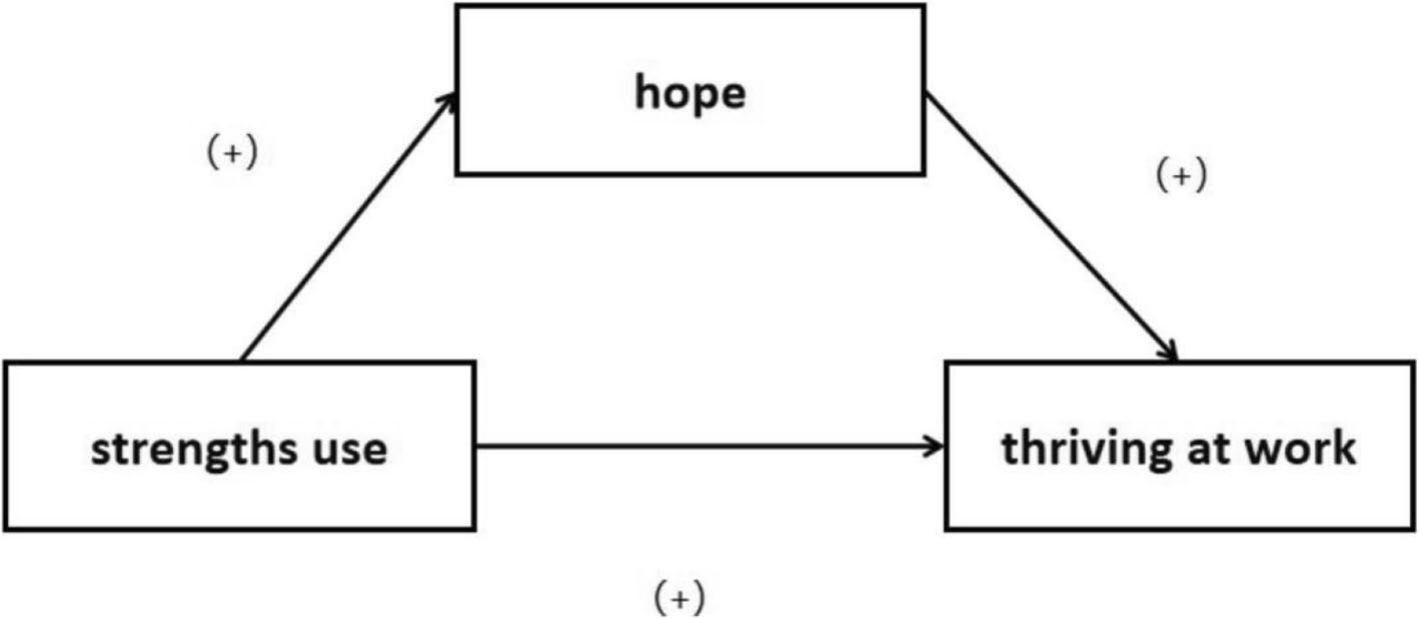
The conceptual model

This study shifted attention to the strengths and resources of RNs, diverging from the dominant focus on the negative aspects of their work in prior research. Drawing upon established literature, a theoretical model was proposed to explore this focus (Fig. 1).

## Materials and methods

### Participants

In this research conducted between January and March 2024, clinical nurses from two tertiary general hospitals in Guangzhou were selected as participants through convenience sampling.Data were collected using an anonymous self-reported questionnaire. A link to the electronic questionnaire was sent to the nurse manager via WeChat, with the approval of the Nursing Department, and then distributed to the clinical nurses. Two uniformly trained investigators continuously monitored the recovery data. When no new data were generated for a consecutive week, the data were exported. A total of 600 questionnaires were completed; however, we excluded 32 surveys due to missing items, completion times of less than 120 s, or identical responses to all items. This resulted in 568 valid responses for analysis.The inclusion criteria were:(1) possession of a valid professional qualification certificate for registered nurses; (2) at least one year of nursing experience; (3) absence of cognitive or behavioral disorders; and (4) informed consent to voluntary participation. Exclusion criteria encompassed nurses currently on leave and those from other hospitals undergoing training.

### Sample size

LPA necessitates a minimum sample size of 300 to ensure the robustness and precision of subgroup classification outcomes [40], with an additional allowance for a 20% attrition rate. Accordingly, a sample of 568 participants was deemed appropriate to yield reliable results for LPA-based investigations.

### Ethical statement

In this study, all respondents gave informed consent and it was approved by the Ethics Committee of Guangdong Provincial Hospital of Traditional Chinese Medicine (YE2022-355-01).

### Instruments

#### Demographic information

Based on previous literature [6, 41], the information collected on participants in this study included general demographic information (gender, age, marital status, highest level of education, etc.) and work-related information (title, position, years of service, form of hospital employment, etc.).

#### Strengths Use Scale (SUS)

The SUS, developed by Govindji and Linley [42], consists of 14 items designed to evaluate individual strengths use. A seven-point Likert scale was used (1 = “disagree strongly” to 7 = “agree strongly”), yielding total scores between 14 and 98, where higher scores denote greater strengths use. The scale has been validated among Chinese nurses [43] (Cronbach α = 0.970). In the present study, SUS demonstrated high internal consistency, with a Cronbach’s alpha of 0.940. Additionally, factor analysis revealed that all 14 items had factor loadings exceeding 0.4, confirming the scale’s robust construct validity [42].

#### Adult Dispositional Hope Scale (ADHS)

Hope was assessed using the ADHS, originally developed by Snyder et al. [44] and revised in its Chinese version by Chen, Shen, and Li [45]. The scale comprises 12 items, divided into two dimensions: motivation (willingness) and pathway thinking, each represented by 4 items, with 4 additional items serving as distractors. Responses are rated on a 4-point scale, though the distractor items were excluded from scoring. The total possible score is 32, with higher scores reflecting a greater level of hope. Hu et al. [30] applied the scale to clinical nurses in China and showed good reliability. In this study, the Cronbach’s alpha coefficient for the overall scale was 0.774, with the subscales yielding coefficients of 0.731 and 0.741, respectively. The high factor loadings of the items on their respective dimensions (> 0.4) further confirm the scale’s robust construct validity [46].

#### Thriving at Work Scale (TWS)

The TWS developed by Porath [47] remains the most widely adopted tool for assessing individual work prosperity, with its Chinese version having been validated in prior studies [48]. Comprising two dimensions—learning and vitality—across 10 items, it employs a 7-point Likert scale (1 = “totally disagree” to 7 = “totally agree”), with items 4 and 8 reverse scored. The total score, ranging from 10 to 70, reflects varying levels of work prosperity, with higher scores signifying greater well-being at work. Several scholars have applied it to the Chinese nurse population with good applicability and reliability [49]. In this study, the Cronbach’s alpha for the overall scale was 0.822, with subscale reliabilities of 0.737 and 0.774, respectively. All items demonstrated high factor loadings (> 0.4) on their respective dimensions, supporting the scale’s robust construct validity [50].

### Statistical analysis

Descriptive analyses were initially employed to summarize participants’ demographic characteristics and occupational profiles, with Harman’s one-way model utilized to assess the presence of common method variance (CMV) [51]. Pearson correlation analyses subsequently measured the linear relationships among strengths use, hope, and work thriving [52]. LPA, based on SIS scores, was conducted to identify subgroups reflecting varying levels of strengths use, with model fit evaluated using entropy, Akaike (AIC), Bayesian (BIC), and sample size-adjusted BIC (aBIC) indices [53]. Univariate and multivariate analyses further identified factors associated with the derived LPA profiles. Additionally, a Bayesian independent sample t-test was conducted to compare work thriving across LPA profiles. The mediating effect of hope between strengths use and work thriving was tested using SPSS version 26.0 (PROCESS-Model 4). All statistical analyses were performed using SPSS Version 26.0 (IBM, Armonk, NY, USA), Mplus (version 8.3), JASP (0.16.1), and Empower Stats (version 4.1).

## Results

### Sample characteristics

A total of 568 clinical nurses were initially included in this survey. The ratio of men to women was 1:17.32, the average age was 37.2 ± 0.7, 58.1% of the participants were married, the number of people with a bachelor’s degree was 91.55%, and 81.51% of them were contractual employees. The average level of thriving at work was 50.09 ± 8.804.

### Common method variance test

Harman’s one-way factor analysis was used to identify the presence of common method bias, and an exploratory factor analysis was conducted on all entries of the group of clinical nurses’ strengths use, hopefulness traits, and work prosperity, and the factors were extracted using the principal component approach. The results showed that there were a total of 7 factors with eigenroots > 1, and the variance explained by the 1st factor was 38.88%, which was < 40% of the critical value, indicating that there was no serious problem of common bias in this study.

### Pearson’s analysis of the correlation between strengths use, hope and thriving at work

Strengths use demonstrated a significant positive correlation with both hope (*r* = 0.493, *P* < 0.001) and thriving at work (*r* = 0.781, *P* < 0.001), while hope also exhibited a positive correlation with thriving at work (*r* = 0.530, *P* < 0.001). Detailed results were provided in Table 1. To examine potential multicollinearity among the independent variables, variance inflation factors (VIF) and tolerance tests were conducted. Multicollinearity is suggested when VIF exceed 10 or tolerance falls below 0.1. In this analysis, VIF values remained below 10 and tolerance values above 0.1 for all variables, indicating no significant multicollinearity concerns. Further specifics were outlined in Table 2.

**Table 1:** Correlates of strengths use, hope and thriving at work among nurses(r)

Variables	Strengths use	Hope	Thriving at work
Strengths use	1		
Hope	0.493**	1	
Thriving at work	0.781**	0.530**	1

** *P* < 0.001

**Table 2:** Correlations (Outcome variable: thriving at work)

Variables	Beta	t	P	TOL	VIF
Constant	——	9.337	< 0.001	——	——
Strengths use	0.687	23.596	< 0.001	0.757	1.321
Hope	0.191	6.572	< 0.001	0.757	1.321

### Latent profile analysis of strengths use

**Fig. 2:**
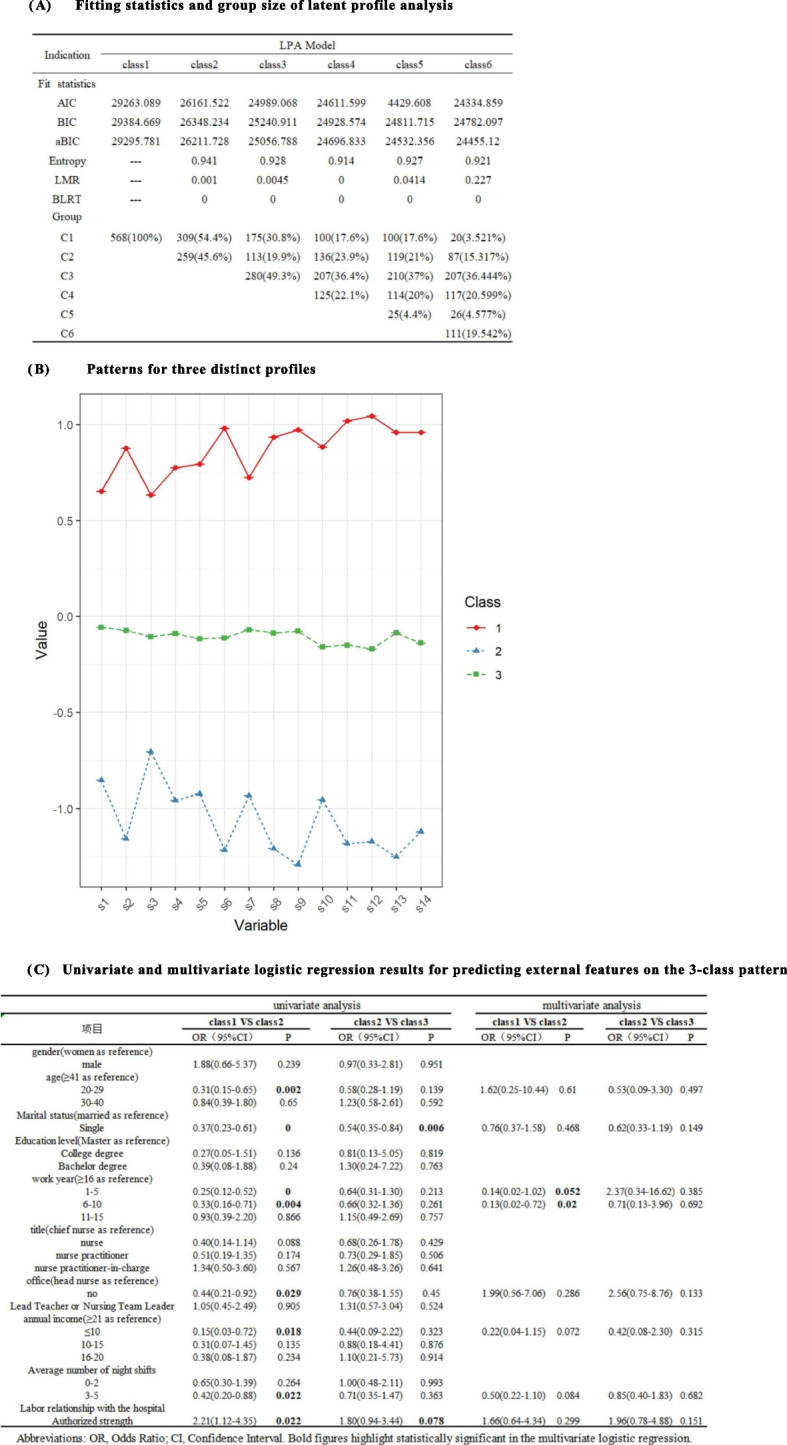
**A** Fitting statistics and group size of latent profile analysis. **B** Patterns for three distinct profiles. **C** Univariate and multivariate logistic regression results for predicting external features on the 3-class pattern

A total of 1 to 6 latent category models were explored after standardisation of the 568 nurses’ advantage use scores for latent feature analysis. The results of the model fit metrics are shown in Fig. 2A. Considering the proportions of each model and the practical significance of the results, we found the 3-profile model to be the most appropriate model. Figure 2B shows the LPA-based 3-profile model. The first profile includes individuals with low use of dominance (30.8%, *N* = 175), the second profile includes individuals with moderate use of dominance (19.9%, *N* = 240), and the third profile includes individuals with high use of dominance (49.3%, *N* = 49). By logistic regression (see Fig. 2C.), after controlling for covariates, only labour relations with the hospital were indicative of strengths use (OR = 2.21, 95% CI: 1.12–4.35, *p* = 0.022; OR = 1.80, 95% CI: 0.94–3.44, *p* = 0.078).

### LPA-based strengths use differences on thriving at work scores

**Fig. 3:**
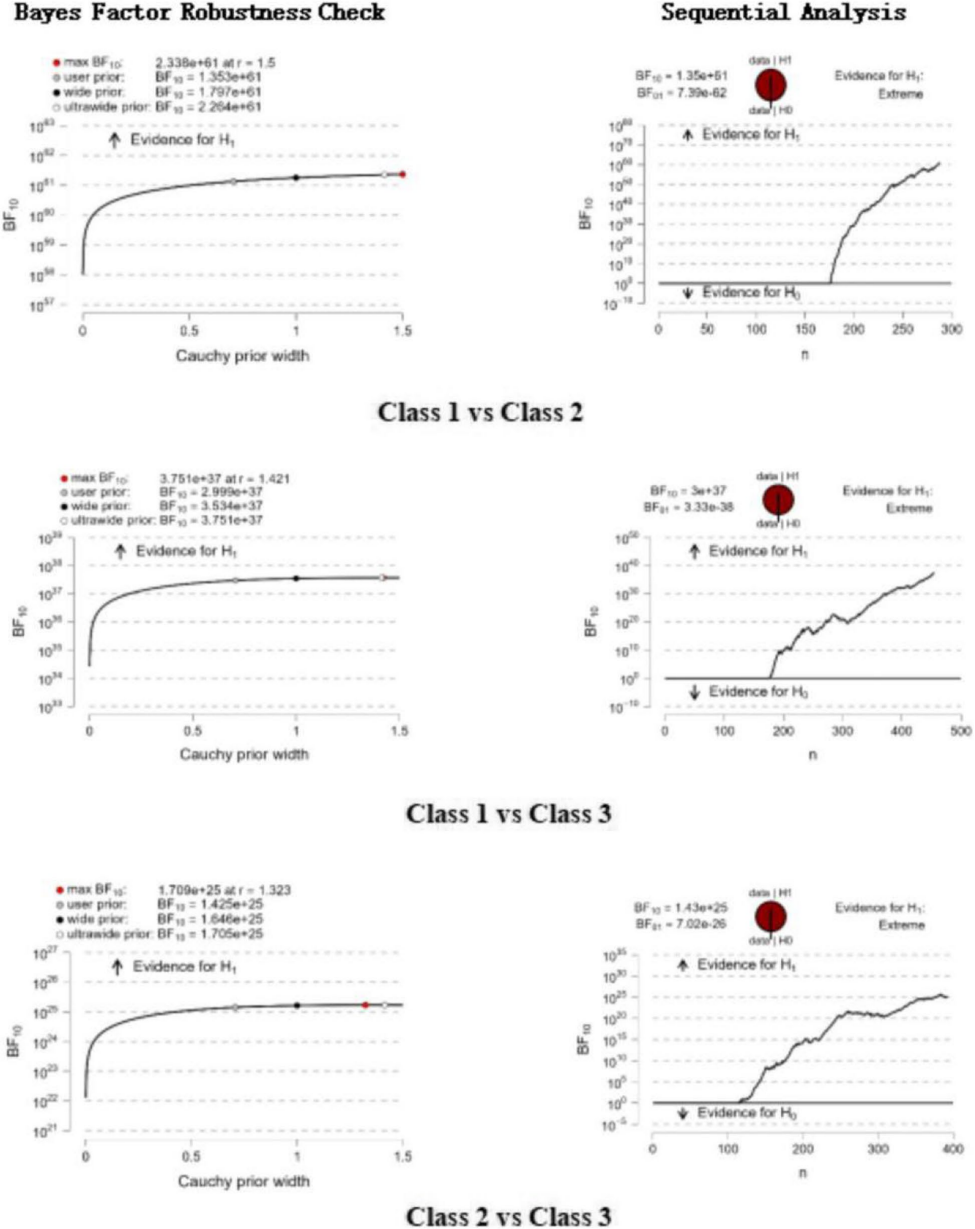
Bayesian factor robustness checks and Bayesian factor sequence analysis based on three different profiles

Significant differences in thriving at work were observed between ‘high speciality use’ and ‘low speciality use’ (BF10 = 1.35e + 61), ‘high speciality use’ and ‘medium speciality use’ (BF10 = 3e + 37), as well as between ‘low speciality use’ and ‘medium speciality use’ (BF10 = 1.43e + 25). Bayesian factor robustness analyses corroborated these distinctions, with further details illustrated in Fig. 3.

### Mediation analysis of hope between LPA-based strengths use and thriving at work

**Fig. 4:**
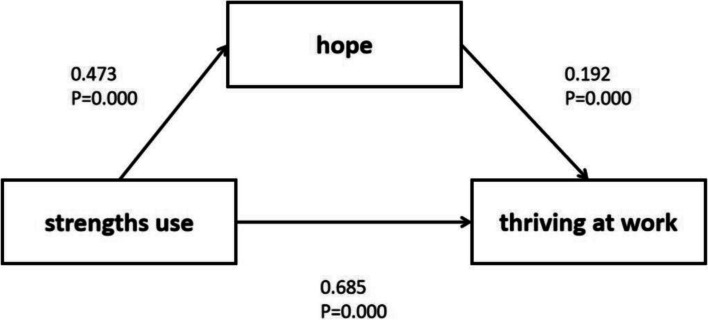
A hypothetical mediator model with three LPA-based types of strengths use as the independent variable (X), hope as the mediator variable (M), and thrving at work as the dependent variable (Y). The common type is used as a reference. Control variables are not shown in the figure for brevity

After adjusting for general variables and designating “moderately strengths use” as the reference group, the analysis revealed the following 95% bootstrap confidence intervals for the effects (Table 3): indirect (-4.04, -2.19), direct (-15.93, -12.74), and total (-18.91, -15.91). The mediating role of hope was found to be significant, demonstrating full mediation between “moderately strengths use” and “highly strengths use.” Furthermore, when “higher strengths use” was set as the reference group, the analysis yielded indirect (-1.98, -0.95), direct (-8.56, -6.20), and total (-10.01, -7.62) effects, confirming a significant full mediation between “lower strengths use” and “higher strengths use.” The mediating effect of hope was also significant in the relationship between “low strengths use” and “high strengths use.” The details are shown in Fig. 4.

**Table 3:** The mediation effect of hope between strengths use and thriving at work among nurses

Direct and indirect effect of strengths use on thriving at work (Middle strengths use as reference)						
	Variables	Effect	SE	t	LLCI	ULCI
Total effects	High strengths use	−17.4104	0.7629	−22.8222	−18.9088	−15.912
	Low strengths use	−8.815	0.6091	−14.4711	−10.0115	−7.6185
Direct effects	High strengths use	−14.335	0.8112	−17.6706	−15.9284	−12.7416
	Low strengths use	−7.3828	0.6012	−12.2799	−8.5637	−6.2019
Indirect effects	High strengths use	−3.0754	0.4675	–-	−4.0348	−2.1913
	Low strengths use	−1.4322	0.2622	–-	−1.9837	−0.9447

## Discussion

This study examined the interplay between strengths use, hope, and job thriving, alongside the variability in strengths use within a sample of 568 clinical nurses from two tertiary hospitals in Guangzhou City. All hypotheses were confirmed. First, findings indicated a positive association between strengths use and job thriving, consistent with prior research [25]. This link is attributable to the increased vigor and task engagement observed in individuals utilizing their strengths at work [54]. Moreover, employees who capitalize on their strengths tend to rapidly acquire relevant skills and knowledge [55], reinforcing earlier findings that strength utilization enhances personal learning [56]. Since thriving at work involves both learning and vigor, a positive relationship between strengths use and job thriving is a logical conclusion. This emphasizes the significance of integrating strengths-based strategies in healthcare environments. Nursing leaders are advised to promote the identification and active application of strengths to cultivate workplace flourishing.

In alignment with the second hypothesis, LPA categorized the sample into three distinct profiles: low, medium, and high strengths use. Approximately half of the nurses demonstrated a moderate level of strengths use, aligning with results from earlier studies [43]. Organizational cultures often prioritize addressing employees’ weaknesses rather than leveraging their strengths [57], potentially constraining the utilization of nurses’ personal strengths. Comparative group analysis revealed that married nurses exhibited higher strengths use than their single counterparts, consistent with previous research indicating that married nurses displayed greater creativity in their work [58]. Marriage is commonly linked to emotional maturity, with married nurses often benefiting from more stable family environments and enhanced emotional support [59], contributing to greater self-confidence and a stronger inclination to leverage their strengths in the workplace. A comparison between different experience levels reveals that the longer the tenure, the higher the degree of strengths use. Extended periods of work experience drive continuous improvement in both professional skills and personal attributes. As these competencies advance, individuals are better equipped to apply their strengths effectively in task execution, leading to increased strengths use. Furthermore, job position plays a significant role in influencing strengths use among nurses. Those without designated positions tend to fall into the lower category of strengths use. This disparity arises because nurses with job titles generally possess greater authority and access to more resources within the care team, offering them more opportunities to apply their strengths. Conversely, nurses without formal positions often face time constraints and are confined to basic nursing tasks, limiting their initiative and creativity in engaging in diverse activities, thereby restricting their potential to demonstrate strengths [60].Thus, a favorable work environment, stable interpersonal relationships, and excellent nursing skills may be key to enhancing nurse advantage use. In the future, nursing education and administrators may develop intervention programs targeting nurse advantage use in these areas.

The third hypothesis is supported by the mediating effect of hope, demonstrating that hope significantly mediates the relationship between strength utilization and job prosperity. This insight broadens the application of the JD-R model and highlights hope’s role in navigating work demands and shaping work outcomes. According to the job demand-resource theory [61], individuals derive benefits from physical, psychological, organizational, or social resources at work, which enhance energy and indirectly improve well-being [62, 63]. Leveraging personal strengths contributes to the development of psychological resources like hope. Nurses with high trait hope exhibit a strong sense of intrinsic motivation, which fosters greater work prosperity. This result is consistent with prior studies identifying hope as a valuable psychological resource positively influencing nurses’ work conditions [64]. Therefore, raising nurses’ hopes should be incorporated into nursing management to effectively reduce the likelihood of nurses leaving their current jobs.

### Practical implications and recommendations

This study outlines strategies to foster employee development within healthcare settings, emphasizing the importance of understanding varying degrees of strengths use among nurses. Nurses identified as ‘low strengths use’ (C2) often face issues such as reduced job satisfaction and unclear self-perception [65], requiring targeted managerial attention. To address this, organizations can encourage strengths identification through the “best self” methodology [43]. For nurses categorized as having a ‘moderate level of strengths use’ (C3), appropriate task delegation and the creation of opportunities to maximize their potential are essential to promote proactive strengths engagement. Nurses exhibiting ‘high strengths use’ (C1) effectively leverage their abilities in the workplace, suggesting that core psychological needs, including autonomy, competence, and relatedness, are being fulfilled, allowing for a stable and positive work attitude. Providing consistent positive feedback and recognition can further support these individuals in advancing their professional trajectories. Additionally, hope is shaped by elements such as rewards, working conditions, and management styles, which serve as key sources of support, helping nurses derive spiritual fulfillment and thereby elevating their sense of hope [66]. Nursing managers must prioritize addressing both the psychological and material needs of nurses through tailored emotional support, spiritual motivation, and adequate rewards [38, 67, 68]. This could involve introducing psychological counseling and stress management initiatives, creating transparent promotion pathways, and improving compensation and benefits structures. Furthermore, psychological capital development programs—such as mental health education and positive thinking interventions—are essential for fostering hope and improving nurses’ performance in the workplace [69, 70].

### Limitations

There are some limitations of this study. This study was conducted in two tertiary-level Chinese hospitals in Guangzhou, and the convenience sampling method may not be fully representative of nurses from different regions and organisations, affecting the generalisation of the current results to all nurses. Indeed, although the sample was fairly representative in terms of gender, age and wards, future studies could be conducted with larger samples and could also investigate these profiles in different regions as well as different cultures and countries to explore possible differences and commonalities. Secondly, it is difficult to generalise the results as our data were collected through self-report and there is a social desirability bias. Finally, we recognise the limitations of cross-sectional designs in identifying causal relationships and plan to use longitudinal designs in future studies to better understand the temporal order and causal relationships between variables.

## Conclusion

The study conclusively identified a positive association between strengths use and nurses’ work flourishing, with hope acting as a mediating variable in this dynamic. Furthermore, the heterogeneity in LPA-based strengths use among nurses revealed distinct patterns of advantage use across different groups. These observations provide critical guidance for healthcare professionals and policymakers, emphasizing the need to encourage strengths-based practices and design interventions that cultivate hope. Such targeted initiatives are integral to improving the overall well-being of nurses, ultimately benefiting the wider healthcare system.

## Data Availability

The data that support the findings of this study are available from the corresponding author upon reasonable request.

## References

[1] Cai Y, Li Q, Cao T, Wan Q. Nurses’ work engagement: the influences of ambidextrous leadership, clinical nurse leadership and workload. J Adv Nurs. 2023;79(3):1152–61. 10.1111/jan.15086. 34723406 10.1111/jan.15086

[2] El-Gazar HE, Abdelhafez S, Zoromba MA. Effects of the areas of worklife on job embeddedness: a national cross-sectional study among Egyptian nurses. BMC Nurs. 2022;21(1):353. 10.1186/s12912-022-01107-6. 36510228 10.1186/s12912-022-01107-6 PMC9742651

[3] Cao J, Jia Z, Zhu C, Li Z, Liu H, Li F, Li J. Nurses’ turnover intention and associated factors in general hospitals in China: a cross-sectional study. J Nurs Manag. 2021;29(6):1613–22. 10.1111/jonm.13295. 33639014 10.1111/jonm.13295

[4] Najafi Z, Sadat-Hoseini A-S, Imanipour M, Mosadeghrad AM. Factors affecting nurses’ retention in Iranian hospitals. J Nurs Manag. 2022;30(3):785–94. 10.1111/jonm.13568. 35218597 10.1111/jonm.13568

[5] Liu D, Zhang S, Wang Y, Yan Y. The antecedents of thriving at work: a meta-analytic review. Front Psychol. 2021;12. 10.3389/fpsyg.2021.659072. 10.3389/fpsyg.2021.659072 PMC8374041 34421715

[6] Zhu X, Kunaviktikul W, Sirakamon S, Abhicharttibutra K, Turale S. A causal model of thriving at work in Chinese nurses. Int Nurs Rev. 2021;68(4):444–52. 10.1111/inr.12671. 33682932 10.1111/inr.12671

[7] Moloney W, Fieldes J, Jacobs S. An integrative review of how healthcare organizations can support hospital nurses to thrive at work. Int J Environ Res Public Health. 2020;17(23):8757. 10.3390/ijerph17238757. 33255725 10.3390/ijerph17238757 PMC7728312

[8] Mortier AV, Vlerick P, Clays E. Authentic leadership and thriving among nurses: the mediating role of empathy. J Nurs Manag. 2016;24(3):357–65. 10.1111/jonm.12329. 26264773 10.1111/jonm.12329

[9] Ren Z, Zhou C, Zhang X, Yang A, Li W, Liu H. Emotional labor, fatigue, and presenteeism in Chinese nurses: the role of organizational identification. BMC Nurs. 2024;23(1):673. 10.1186/s12912-024-02351-8. 39304888 10.1186/s12912-024-02351-8 PMC11416020

[10] Spreitzer G, Sutcliffe K, Dutton J, Sonenshein S, Grant AM. A socially embedded model of thriving at work. Organ Sci. 2005;16(5):537–49. 10.1287/orsc.1050.0153.

[11] Lin CP, Xian J, Li B, Huang H. Transformational leadership and employees’ thriving at work: the mediating roles of challenge-hindrance stressors. Front Psychol. 2020;11:1400. 10.3389/fpsyg.2020.01400. 32655458 10.3389/fpsyg.2020.01400 PMC7325323

[12] Zhai Y, Cai S, Chen X, Zhao W, Yu J, Zhang Y. The relationships between organizational culture and thriving at work among nurses: the mediating role of affective commitment and work engagement. J Adv Nurs. 2023;79(1):194–204. 10.1111/jan.15443. 36104977 10.1111/jan.15443

[13] Li C, Hou X, Cui X, Zhao Y, Zhu Y. Factors influencing the thriving of emergency department nurses in China. Int Emerg Nurs. 2024;74:101441. 10.1016/j.ienj.2024.101441. 38531212 10.1016/j.ienj.2024.101441

[14] Ilies R, Liu Y, Aw S, Las Heras M, Rofcanin Y. Why does using personal strengths at work increase employee engagement, who makes the most out of it, and how? J Occup Health Psychol. 2024;29(2):113–29. 10.1037/ocp0000374. 38647463 10.1037/ocp0000374

[15] Hai S, Park I-J. Strengths use for tasks and relationships in organizations: development and validation of a strengths use scale. Front Psychol. 2022;13:659046. 10.3389/fpsyg.2022.659046. 35386899 10.3389/fpsyg.2022.659046 PMC8979025

[16] van Woerkom M, Oerlemans W, Bakker AB. Strengths use and work engagement: a weekly diary study. Eur J Work Organ Psy. 2016;25(3):384–97. 10.1080/1359432X.2015.1089862.

[17] Taylor EC, Livingston LA, Clutterbuck RA, Callan MJ, Shah P. Psychological strengths and well-being: strengths use predicts quality of life, well-being and mental health in autism. Autism. 2023;27(6):1826–39. 10.1177/13623613221146440. 36639858 10.1177/13623613221146440 PMC10375006

[18] Jin W, Zheng X, Gao L, Cao Z, Ni X. Basic psychological needs satisfaction mediates the link between strengths use and teachers’ work engagement. Int J Environ Res Public Health. 2022;19(4):2330. 10.3390/ijerph19042330. 35206518 10.3390/ijerph19042330 PMC8872018

[19] Demir OB, Yilmaz FT. The effect of posture regulation training on musculoskeletal disorders, fatigue level and job performance in intensive care nurses. BMC Nurs. 2024;23(1):778. 10.1186/s12912-024-02387-w. 39438895 10.1186/s12912-024-02387-w PMC11498948

[20] Deci EL, Ryan RM. The general causality orientations scale: self-determination in personality. J Res Pers. 1985;19(2):109–34. 10.1016/0092-6566(85)90023-6.

[21] El-Gazar HE, Taie ES, Elamir H, Abou Zeid MAG, Magdi HM, Zoromba MA. Does the presence of calling relate to career success? The role of strengths use and deficit correction among nurses. Int Nurs Rev. 2024;71(4):823–31. 10.1111/inr.12924. 38174920 10.1111/inr.12924

[22] Bakker A, Hetland J, (Jørn), Kjellevold-Olsen, O., & Espevik R. Daily strengths use and employee well-being: the moderating role of personality. J Occup Organ Psychol. 2019;92:144–68. 10.1111/joop.12243.

[23] Ding H, Chu X. Employee strengths use and thriving at work: the roles of self-efficacy and perceived humble leadership. J Pers Psychol. 2020;19(4):197–205. 10.1027/1866-5888/a000262.

[24] Bai C, Bai B, Yang J, Zhou S. Perceived organizational support for strengths use and its impact on nurses’ job performance: the mediating roles of control beliefs about stress and optimism. Int Nurs Rev. 2024. 10.1111/inr.13028. 10.1111/inr.13028 39046241

[25] Chu X, Zhang L, Li M. Nurses’ strengths use and turnover intention: the roles of job crafting and self-efficacy. J Adv Nurs. 2022;78(7):2075–84. 10.1111/jan.15124. 34859903 10.1111/jan.15124

[26] Yang Q, Zhao A, Lee C, Wang X, Vorderstrasse A, Wolever RQ. Latent profile/class analysis identifying differentiated intervention effects. Nurs Res. 2022;71(5):394–403. 10.1097/NNR.0000000000000597. 35417442 10.1097/NNR.0000000000000597

[27] Snyder CR. Hope theory: rainbows in the mind. Psychol Inq. 2002;13(4):249–75. 10.1207/S15327965PLI1304_01.

[28] Broetje S, Jenny GJ, Bauer GF. The key job demands and resources of nursing staff: an integrative review of reviews. Front Psychol. 2020;11:84. 10.3389/fpsyg.2020.00084. 32082226 10.3389/fpsyg.2020.00084 PMC7005600

[29] Peng X, Wu D. The protective effect of grit on clinical nurses’ occupational psychological distress: mediating and suppressing effects of Hope. Front Psychol. 2022;13:1019655. 10.3389/fpsyg.2022.1019655. 36248447 10.3389/fpsyg.2022.1019655 PMC9559393

[30] Hu H, Wang C, Lan Y, Wu X. Nurses’ turnover intention, hope and career identity: The mediating role of job satisfaction. BMC Nurs. 2022;21(1):43. 10.1186/s12912-022-00821-5. 35144604 10.1186/s12912-022-00821-5 PMC8830989

[31] Katajisto M, Hyvärinen S, Uusiautti S. Changes in Finnish ninth graders’ positive psychological capital (PsyCap) in a strength-based student guidance intervention. Int J Adolesc Youth. 2021;26(1):321–39. 10.1080/02673843.2021.1943469.

[32] Green ZA. Character strengths intervention for nurturing well-being among Pakistan’s university students: a mixed-method study. Appl Psychol Health Well Being. 2022;14(1):252–77. 10.1111/aphw.12301. 34431238 10.1111/aphw.12301

[33] Bai C, Bai B. Strength use and workers’ job burnout in the centers for disease control and prevention: the mediating role of psychological capital. J Adv Nurs. 2023;79(6):2328–36. 10.1111/jan.15586. 36762675 10.1111/jan.15586

[34] Zhang F, Zhao L, Zeng Y, Xu K, Wen X. A comparison of inquiry-oriented teaching and lecture-based approach in nursing ethics education. Nurse Educ Today. 2019;79:86–91. 10.1016/j.nedt.2019.05.006. 31108384 10.1016/j.nedt.2019.05.006

[35] El-Gazar HE, Zoromba MA. Ethical leadership, flourishing, and extra-role behavior among nurses. SAGE Open Nurs. 2021;7:23779608211062668. 10.1177/23779608211062669. 10.1177/23779608211062669 PMC8832327 35155773

[36] Flinkman M, Rudman A, Pasanen M, Leino-Kilpi H. Psychological capital, grit and organizational justice as positive strengths and resources among registered nurses: a path analysis. Nurs Open. 2023;10(8):5314–27. 10.1002/nop2.1769. 37128977 10.1002/nop2.1769 PMC10333875

[37] Yan D, Wen F, Li X, Zhang Y. The relationship between psychological capital and innovation behaviour in Chinese nurses. J Nurs Manag. 2020;28(3):471–9. 10.1111/jonm.12926. 31811781 10.1111/jonm.12926

[38] Alan H, Polat S, Tiryaki Sen H. The role of psychological capital in the relationship between nurses’ job satisfaction and turnover intention. Perspect Psychiatr Care. 2022;58(4):2811–9. 10.1111/ppc.13128. 35726709 10.1111/ppc.13128

[39] Flinkman M, Coco K, Rudman A, Leino-Kilpi H. Registered nurses’ psychological capital: a scoping review. Int J Nurs Pract. 2023;29(5):e13183. 10.1111/ijn.13183. 37485748 10.1111/ijn.13183

[40] Sinha P, Calfee CS, Delucchi KL. Practitioner’s guide to latent class analysis: methodological considerations and common pitfalls. Crit Care Med. 2021;49(1):e63–79. 10.1097/CCM.0000000000004710. 33165028 10.1097/CCM.0000000000004710 PMC7746621

[41] Yun Z, Zhou P, Zhang B. High-performance work systems, thriving at work, and job burnout among nurses in Chinese public hospitals: the role of resilience at work. Healthcare (Basel, Switzerland). 2022;10(10):1935. 10.3390/healthcare10101935. 36292382 10.3390/healthcare10101935 PMC9601832

[42] Govindji R, Linley PA. Strengths use, self-concordance and well-being: implications for strengths coaching and coaching psychologists. Int Coaching Psychol Rev. 2007;2(2):143–153. 10.53841/bpsicpr.2007.2.2.143.

[43] Bai C, Bai B, Kong F. Strength use and nurses’ depressive symptoms: the mediating role of basic psychological needs satisfaction. J Nurs Manag. 2021;29(6):1660–7. 10.1111/jonm.13322. 33792987 10.1111/jonm.13322

[44] Snyder CR, Harris C, Anderson JR, Holleran SA, Irving LM, Sigmon ST, Yoshinobu L, Gibb J, Langelle C, Harney P. The will and the ways: Development and validation of an individual-differences measure of hope. J Pers Soc Psychol. 1991;60(4):570–85. 10.1037/0022-3514.60.4.570. 2037968 10.1037//0022-3514.60.4.570

[45] DiGasbarro D, Midden A, Van Haitsma K, Meeks S, Mast B. Reliability and validity of the adult hope scale among nursing home residents with and without cognitive impairment. Clin Gerontol. 2020;43(3):340–9. 10.1080/07317115.2019.1656696. 31453758 10.1080/07317115.2019.1656696 PMC7133019

[46] Sun Q, Ng KM, Wang C. A validation study on a new Chinese version of the dispositional hope scale. Meas Eval Couns Dev. 2012;45:133–48. 10.1186/s41155-023-00246-2.

[47] Porath C, Spreitzer G, Gibson C, Garnett FG. Thriving at work: toward its measurement, construct validation, and theoretical refinement. J Organ Behav. 2012;33(2):250–75. 10.1002/job.756.

[48] de Beer J, Rawas H, Beheri W. Workplace dignity amongst clinical nurses. BMC Nurs. 2024;23(1):715. 10.1186/s12912-024-02376-z. 39369242 10.1186/s12912-024-02376-z PMC11452934

[49] Li F, Zhou Y, Kuang P. Thriving at work, career calling, and moral distress among nurses. Nurs Ethics. 2024;31(5):919–29. 10.1177/09697330231215948. 38116631 10.1177/09697330231215948

[50] Peters SE, Gundersen DA, Katz JN, Sorensen G, Wagner GR. Thriving from work questionnaire: dimensionality, reliability, and validity of the long and short form questionnaires. Am J Ind Med. 2023;66(4):281–96. 10.1002/ajim.23465. 36748853 10.1002/ajim.23465 PMC13048217

[51] Beasley TM. Tests of mediation: paradoxical decline in statistical power as a function of mediator collinearity. J Exp Educ. 2014;82(3):283–306. 10.1080/00220973.2013.813360. 24954952 10.1080/00220973.2013.813360 PMC4061717

[52] Wagner-Łosieczka B, Kolarczyk E, Młynarska A, Owczarek D, Sadowski M, Kowalczuk K, Guzak B, Czapla M, Uchmanowicz I. The variables in the rationing of nursing care in cardiology departments. BMC Nurs. 2023;22(1):59. 10.1186/s12912-023-01222-y. 36869327 10.1186/s12912-023-01222-y PMC9983219

[53] Tein J-Y, Coxe S, Cham H. Statistical power to detect the correct number of classes in latent profile analysis. Struct Equ Modeling. 2013;20(4):640–57. 10.1080/10705511.2013.824781. 24489457 10.1080/10705511.2013.824781 PMC3904803

[54] van Woerkom M, Mostert K, Els C, Bakker AB, de Beer L, Rothmann S Jr. Strengths use and deficit correction in organizations: development and validation of a questionnaire. Eur J Work Organ Psy. 2016;25(6):960–75. 10.1080/1359432X.2016.1193010.

[55] Bartz D. Managers effectively applying strengths management and emotional intelligence. Int J Humanit Soc Sci. 2018;8. 10.30845/IJHSS.V8N10P1.

[56] Galloway R, Reynolds B, Williamson J. Strengths-based teaching and learning approaches for children. In: Fan, S., Fielding-Wells, J. (eds) What is Next in Educational Research?. SensePublishers, Rotterdam. 2016. 10.1007/978-94-6300-524-1_19.

[57] Miglianico M, Dubreuil P, Miquelon P, et al. Strength use in the workplace: a literature review. J Happiness Stud. 2020;21:737–64. 10.1007/s10902-019-00095-w.

[58] Tsai H-M, Liou S-R, Hsiao Y-C, Cheng C-Y. The relationship of individual characteristics, perceived worksite support and perceived creativity to clinical nurses’ innovative outcome. J Clin Nurs. 2013;22(17–18):2648–57. 10.1111/jocn.12269. 23710647 10.1111/jocn.12269

[59] Yu J, Song H, Shi H, Wang K. Association between work-family conflict and overall well-being among Chinese nurse leaders. J Nurs Manag. 2020;28(7):1498–503. 10.1111/jonm.13084. 32629527 10.1111/jonm.13084

[60] Grealish L, Hyde MK, Legg M, Lazenby M, Aitken JF, Dunn J, Chambers SK. Psychosocial predictors of hope two years after diagnosis of colorectal cancer: implications for nurse-led hope programmes. Eur J Cancer Care. 2019;28(3):e13010. 10.1111/ecc.13010. 10.1111/ecc.13010 30740814

[61] Bakker AB, Demerouti E. Job demands-resources theory: taking stock and looking forward. J Occup Health Psychol. 2017;22(3):273–85. 10.1037/ocp0000056. 27732008 10.1037/ocp0000056

[62] Bakker AB, Demerouti E. The job demands-resources model: State of the art. J Manag Psychol. 2007;22(3):309–28. 10.1108/02683940710733115.

[63] Pansini M, Buonomo I, De Vincenzi C, Ferrara B, Benevene P. Positioning technostress in the JD-R model perspective: a systematic literature review. Healthcare (Basel, Switzerland). 2023;11(3):446. 10.3390/healthcare11030446. 36767021 10.3390/healthcare11030446 PMC9914396

[64] Ong NY, Teo FJJ, Ee JZY, Yau CE, Thumboo J, Tan HK, Ng QX. Effectiveness of mindfulness-based interventions on the well-being of healthcare workers: a systematic review and meta-analysis. General Psychiatry. 2024;37(3):e101115. 10.1136/gpsych-2023-101115. 38737894 10.1136/gpsych-2023-101115 PMC11086195

[65] Zhang Y, Chen M. Character strengths, strengths use, future self-continuity and subjective well-being among Chinese university students. Front Psychol. 2018;9:1040. 10.3389/fpsyg.2018.01040. 30008686 10.3389/fpsyg.2018.01040 PMC6034163

[66] Antunes M, Laranjeira C, Querido A, Charepe Z. “What do we know about hope in nursing care?”: a synthesis of concept analysis studies. Healthcare (Basel, Switzerland). 2023;11(20):2739. 10.3390/healthcare11202739. 37893813 10.3390/healthcare11202739 PMC10606526

[67] Gon R, Wu Y, Liu Y, Yang R, Zhang Y, Xing L. Influence of group training based on psychological capital theory on nursing staff’s occupational benefits and job satisfaction in an infusion preparation center. Altern Ther Health Med. 2023;29(3):186–92. 36795521

[68] El-Gazar HE, Abdelhafez S, Ibrahim N, Shawer M, Zoromba MA. Effect of job crafting intervention program on harmonious work passion and career commitment among nurses: a randomized controlled trial. J Nurs Manag. 2023;2023(1):9623866. 10.1155/2023/9623866. 10.1155/2023/9623866 PMC11918986 40225640

[69] Monroe C, Loresto F, Horton-Deutsch S, Kleiner C, Eron K, Varney R, Grimm S. The value of intentional self-care practices: the effects of mindfulness on improving job satisfaction, teamwork, and workplace environments. Arch Psychiatr Nurs. 2021;35(2):189–94. 10.1016/j.apnu.2020.10.003. 33781399 10.1016/j.apnu.2020.10.003 PMC7553100

[70] Ma H, Zhu X, Huang J, Zhang S, Tan J, Luo Y. Assessing the effects of organizational support, psychological capital, organizational identification on job performance among nurses: a structural equation modeling approach. BMC Health Serv Res. 2023;23(1):806. 10.1186/s12913-023-09705-z. 37501166 10.1186/s12913-023-09705-z PMC10375763

